# Proteomic Analysis Dissects Molecular Mechanisms Underlying Plant Responses to Phosphorus Deficiency

**DOI:** 10.3390/cells11040651

**Published:** 2022-02-14

**Authors:** Ming Zhou, Shengnan Zhu, Xiaohui Mo, Qi Guo, Yaxue Li, Jiang Tian, Cuiyue Liang

**Affiliations:** 1 Root Biology Center, State Key Laboratory for Conservation and Utilization of Subtropical Agro-Bioresources, College of Natural Resources and Environment, South China Agricultural University, Guangzhou 510642, China; 1476500801@stu.scau.edu.cn (M.Z.); xhmo@scau.edu.cn (X.M.); guoqi1421@163.com (Q.G.); yaxueli2021@163.com (Y.L.); 2 Life Science and Technology School, Lingnan Normal University, Zhanjiang 524048, China; shnzhu@163.com

**Keywords:** phosphorus, proteomics, P use efficiency

## Abstract

Phosphorus (P) is an essential nutrient for plant growth. In recent decades, the application of phosphate (Pi) fertilizers has contributed to significant increases in crop yields all over the world. However, low efficiency of P utilization in crops leads to intensive application of Pi fertilizers, which consequently stimulates environmental pollution and exhaustion of P mineral resources. Therefore, in order to strengthen the sustainable development of agriculture, understandings of molecular mechanisms underlying P efficiency in plants are required to develop cultivars with high P utilization efficiency. Recently, a plant Pi-signaling network was established through forward and reverse genetic analysis, with the aid of the application of genomics, transcriptomics, proteomics, metabolomics, and ionomics. Among these, proteomics provides a powerful tool to investigate mechanisms underlying plant responses to Pi availability at the protein level. In this review, we summarize the recent progress of proteomic analysis in the identification of differential proteins that play roles in Pi acquisition, translocation, assimilation, and reutilization in plants. These findings could provide insights into molecular mechanisms underlying Pi acquisition and utilization efficiency, and offer new strategies in genetically engineering cultivars with high P utilization efficiency.

## 1. Introduction

Phosphorus (P) is an essential mineral nutrient for plants, accounting for up to 0.5% of plant dry weight depending on plant species [1]. It is not only an indispensable structural constituent of biomolecules, including deoxyribonucleic acid (DNA), proteins, and phospholipids, but also is a key signal factor that functions in mediating the phosphate (Pi)-signaling network [2,3,4,5]. Plants mainly take up P in the form of Pi, including HPO_4_^2−^ and H_2_PO_4_^−^, which is about 0.1–10 μM in soils [6]. Meanwhile, multiple factors exist in soils, limiting Pi availability by directly or indirectly chelating Pi into immobile forms, such as microbial activities and an abundance of cations (e.g., Al^3+^, Fe^2+^, Ca^2+^). For example, in acid soils, Pi easily reacts with Al^3+^ or Fe^2+^ and becomes sparingly soluble forms (i.e., Al-P and Fe-P) [6]. Low Pi availability and fluctuation severely limits crop yield by adversely affecting root growth, photosynthesis, respiration, and energy transduction [7,8]. To meet the requirement of P in crops, millions of tons of Pi fertilizers are profligately applied to farms worldwide every year. However, due to low P utilization efficiency in crops, applied Pi fertilizers are either fixed by soil particles or leached into the biosphere, leading to environmental pollution and biodiversity loss [4,9,10]. Therefore, a deeper understanding of the molecular mechanisms regarding plant tolerance to low Pi availability is required to develop cultivars with high Pi fertilizer utilization efficiency, thus ameliorating the sole reliance on excess Pi fertilizer applications to improve crop yield.

In recent decades, adaptive mechanisms underlying plant responses to P deprivation have been extensively investigated through forward and reverse genetic analysis, with the aid of the application of proteomics, transcriptomics, metabolomics, and ionomics [11,12,13,14]. Among these, proteomic analysis, as a powerful tool, has permitted us to identify numerous differentially accumulated proteins (DAPs) in response to Pi availability, which sheds light on molecular responses of plants to Pi starvation at the protein level, especially at protein modification levels (e.g., phosphorylation, succinylation) [13,15]. For example, a set of DAPs was identified at different P levels in many plant species, such as in Arabidopsis (*Arabidopsis thaliana*) [16,17], maize (*Zea mays*) [18,19], tomato (*Solanum lycopersicum*) [20], rice (*Oryza sativa*) [21,22], barley (*Hordeum vulgare*) [23], and soybean (*Glycine max*) [24,25] (Table 1). In this review, we mainly summarize recent advances in the identification and functional characterization of DAPs in response to Pi starvation in plants through proteomic analysis, and highlight the complex regulatory network underlying Pi acquisition, remobilization, and reutilization at protein levels. Meanwhile, we discuss the advantages and challenges of proteomic analysis to shed light on molecular mechanisms underlying plant adaptations to Pi starvation, and thus contribute to developing crop cultivars with high P efficiency in the future.

## 2. Morphological, Physiological, and Biochemical Responses of Plants to Pi Starvation

Phosphorus deficiency has many deleterious effects on plant growth, as reflected by a significant decrease in plant biomass and yield [4,45,46]. It is well known that plants have evolved a set of morphological, physiological, and molecular strategies to adapt to P deficiency conditions. These adaptive strategies could enhance plant capability to acquire Pi from soils and restrict Pi consumption in plant organisms or increase plant internal Pi reutilization [9,47,48,49]. Among plant morphological responses, modification of root morphology and architecture is generally considered to play a key role in controlling plant Pi acquisition efficiency, such as the formation of shallower root architecture and increases in both lateral root length and root hair density [50,51]. Interestingly, in white lupin and several species of the *Proteaceae* family, the formation of cluster roots termed “proteoid roots” was observed, which could enhance root–soil contact and thus facilitate plants to acquire more Pi from soils [52,53,54]. Accompanied by changes in root morphology and architecture, physiological and biochemical responses in plant roots could strengthen the capability of roots to mobilize sparingly soluble P in soils, including increased organic acid synthesis and exudation to mobilize inorganic-P forms (e.g., Al-P, Fe-P, Ca-P), and enhance root-associated purple acid phosphatase activity to hydrolyze organic-P forms (e.g., phytate-P, ATP) [9,55,56]. Meanwhile, plants could form beneficial symbiosis with soil microorganisms, such as arbuscular mycorrhizal (AM), to improve Pi acquisition [57,58,59,60,61].

In addition to changes in root traits, Pi availability also significantly triggers leaf/shoot morphology remodeling. For example, leaf angles (i.e., erectness, inclination) and tiller numbers are regulated by P deficiency in rice [62,63,64]. Moreover, accompanied by changes in shoot morphology, a group of physiological and biochemical processes in response to P deficiency was also influenced. For example, leaf color turned from green to purple or dark-green in most plants under low-P conditions, which was mainly caused by anthocyanin accumulations, along with the reduced chlorophyll contents [28,29,65]. Meanwhile, increased starch content and decreased sucrose content in leaves were widely observed in most plants [66,67]. Therefore, identification and functional characterization of the DAPs related to morphological, physiological, and biochemical responses in plants could help us to further improve Pi fertilizer utilization efficiency in crops [68,69].

## 3. DAPs Reveal Complex Repones of Plants to P Deficiency

### 3.1. Identification of DAPs in Plant Leaves/Shoots

#### 3.1.1. Differential Proteins Related to Photosynthesis and Carbon Metabolism

**Figure 1:**
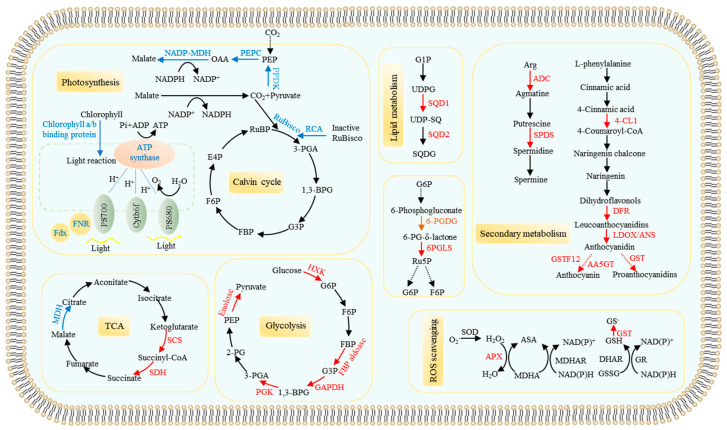
A model of integration of different adaptive strategies to P deficiency regulated by DAPs in shoots or leaves. The DAPs identified by proteomic analysis associated with different metabolic pathways. Red color indicates proteins with increased accumulations in plants under low-P conditions; blue color indicates proteins with decreased accumulations in plants under low-P conditions; brown color suggests proteins exhibiting either up-regulated or down-regulated accumulations under low-P conditions; dashed lines indicate multiple steps; AA5GT, anthocyanidin 5-O-glucosyltransferase; ACC, 1-aminocyclopropane-1-carboxylate; ADP, adenosine diphosphate; ANS, anthocyanidin synthase; APX, ascorbate peroxidase; Arg, arginine; ADC, arginine decarboxylase; ASA, ascorbic acid; ATP, adenosine triphosphate; 1,3-BPG, 1,3-bisphosphoglycerate; 4-CL1, 4-coumaroyl-CoA ligase 1; DFR, dihydroflavonol-4-reductase; DHAR, dehydroascorbate reductase; E4P, erythritos-4-phosphate; F6P, fructose-6-phosphate; FBP aldoase, fructose-1,6-bisphosphate aldolase; FBP, fructose-1,6-bisphosphate; FNR, ferredoxin-nitrite reductase; Fdx, ferredoxin; G1P, glucose-1-phosphate; G3P, glyceraldehyde-3-phosphate; G6P, glucose-6-phosphate; 6-PGDG, 6-phosphogluconate dehydrogenase; GAPDH, glyceraldehyde-3-phosphate dehydrogenase; GR, glutathione reductase; GSH, reduced glutathione; GSSG, oxidized glutathione; GST, glutathione-S-transferase; GSTF12, glutathione-S-transferase F12; HXK, hexokinase; LDOX, leucoanthocyanidin dioxygenase; MDH, malate dehydrogenase; MDHA, monodehydroascorbate; MDHAR, monodehydroascorbate reductase; Met, methionine; NADP-MDH, NADP-malate dehydrogenase; OAA, oxaloacetic acid; PEP, phosphoenolpyruvate; PEPC, phosphoenolpyruvate carboxylase; 2-PG, 2-phosphoglycerate; 3-PGA, 3-phosphoglycerate; 6-PG-δ-lactone, 6-phosphoglucono-δ-lactone; 6PGLS, 6-phosphogluconolactonase; PGK, phosphoglycerate kinase; Pi, phosphate; PPDK, pyruvate orthophosphate dikinase; RCA, ribulose bisphosphate carboxylase/oxygenase activase; Ru5P, Ribulose-5-phosphate; RuBP, Ribulose-1,5-diphosphate; SAM, s-adenosyl methionine; SCS, succinyl-CoA synthetase; SDH, succinate dehydrogenase; SOD, superoxide dismutase; SPDS, spermidine synthase; SQD1, UDP-sulfoquinovose synthase; SQD2, sulfoquinovosyl diacylglycerol synthase; SQDG, sulfoquinovosyl diacylglycerol; TCA cycle, tricarboxylic acid cycle; UDPG, uridine diphosphoglucose; UDP-SQ, UDP-sulfoquinovosyl.

Photosynthesis is a light-harvesting process whose intensity is mainly dependent on the generation rate of NADPH and ATP, as well as ribulose-1,5-diphosphate (RuBP) carboxylation [70,71,72]. Reduced accumulations of proteins associated with the photosynthesis process have been observed in plants under P deficiency conditions through differential proteomic analysis, such as proteins related to light energy absorption, electron transfer, and transformation to generate ATP and NADPH. For example, the differential accumulations of a series of proteins were identified, including chlorophyll a/b binding protein in ramie (*Boehmeria nivea*), ferredoxin (Fdx) in soybean, as well as ferredoxin-nitrite reductase (FNR) and alpha/beta subunit of the ATP synthetase in maize (Figure 1) [18,26,28,29]. Meanwhile, a set of DAPs exhibiting decreased accumulations were found to be involved in rubisco carboxylation reaction (RuBisco), the conversion of ATP and NADPH process, including NADP-malate dehydrogenase (NADP-MDH), pyruvate orthophosphate dikinase (PPDK), phosphoenolpyruvate carboxylase (PEPC), transketolase isoform, RuBisco activase A (RCA), and the large and small subunit of RuBisco [18,26,27,40,41]. Therefore, decreased accumulations of the proteins strongly suggest that Pi starvation could reduce photosynthesis in plants. 

A set of proteins closely related to carbon and energy metabolism were found to be up- or down-regulated by Pi starvation in plant leaves (Figure 1). For example, some proteins associated with the glycolysis process were up-regulated, such as hexokinase (HXK) in ramie leaves, fructose-1,6-bisphosphate aldolase (FBP aldoase), glyceraldehyde-3-phosphate dehydrogenase (GAPDH), phosphoglycerate kinase (PGK), and enolase1 in leaves of both maize and Arabidopsis [18,26,43]. Moreover, the increased accumulations of proteins involved the tricarboxylic acid cycle (TCA) were also found to be affected by Pi deprivation in maize leaves, such as succinyl-CoA synthetase (SCS) and succinate dehydrogenase (SDH) [18]. Additionally, the abundance of MDH and isocitrate dehydrogenase (IDH) was found to be down-regulated in leaves of maize and ramie, respectively [26,27]. Meanwhile, other carbon metabolic processes were also influenced by Pi starvation in plants, as reflected by the identification of related DAPs (Figure 1). For example, the 6-phosphogluconolactonase (6-PGLS) and 6-phosphogluconate dehydrogenase (6-PGDG), associated with the pentose phosphate pathway (PPP), were up-regulated under P deficiency conditions in maize and barley [18,23]. In contrast, triose-phosphate isomerase (TPI) and 6-PGDG were down-regulated in leaves of rape and maize, respectively [18,27,41]. These results indicate that carbon and energy metabolic processes, like glycolysis, TCA cycle, and gluconeogenesis, are significantly influenced in plants under low-P conditions.

#### 3.1.2. Pi Starvation Responsive Proteins Related to Remodeling Lipid Membranes

One of the vital adaptive strategies to low Pi availability in plants is to improve internal P utilization efficiency by remodeling lipid membranes [73]. Under low-Pi stress, phospholipids, accounting for 30% of total organophosphate in plants, were replaced by specific non-phosphorous lipids, such as sulfoquinovosyl diacylglycerol (SQDG) [74]. Consistently, a set of DAPs was identified to function in remodeling lipid membranes. For example, in maize and Arabidopsis leaves, increased accumulations of UDP-sulfoquinovose synthase (SQD1) were observed, which were suggested to function in releasing sulfoquinovose for SQDG production [18,27,75]. Meanwhile, increased accumulations of SQDG synthase (SQD2) were also observed under low-P conditions, and SQDs were suggested to accelerate the replacement of phospholipid glycerate (PG) by SQDG in plant cell plasma membranes [17,75]. Additionally, some proteins, including lipid-transfer protein, lipoxygenase, and 3-phosphoglycerate dehydrogenase, were also observed to be up-regulated in Arabidopsis leaves [17]. These results support the hypothesis that changes in the phospholipid metabolism could further accelerate the conversion of intracellular organic P forms, and thus improve P utilization efficiency under low-P conditions. 

#### 3.1.3. Proteins Involved Anthocyanin, Polyamine, and Reactive Oxygen Species (ROS) Metabolisms

It is generally believed that increases in anthocyanin synthesis in shoots or leaves could protect plants from ROS damage under low-P conditions [76,77,78,79]. Anthocyanins are products generated from the phenylpropanoid and flavonoid metabolic pathways, regulated by multiple enzymes, such as chalcone synthase (CHS) and dihydroflavonol-4-reductase (DFR) [80]. DAPs functioning in anthocyanin synthesis have been found in Arabidopsis leaves through proteomic analysis, including 4-coumarate:CoA ligase 1 (4-CL1), DFR, leucoanthocyanidin dioxygenase (LDOX), anthocyanidin synthase (ANS), anthocyanidin 5-O-glucosyltransferase (AA5GT), phenylalanine ammonia-lyases (PALs), and glutathione-S-transferase F12 (GSTF12) [17,75] (Figure 1). The results strongly suggest that P deficiency could increase accumulations of enzymes functioning in anthocyanin synthesis, and thus enhance anthocyanin accumulations in leaves. 

Polyamines are aliphatic amines and play an important role in plant adaptation to biotic and abiotic stresses [81]. Interestingly, several DAPs controlling polyamines biosynthesis were found in maize leaves under P deficiency conditions, such as spermidine synthase (SPDS) and arginine decarboxylase (ADC) [18]. Although the detailed functions of polyamines in plant adaptation to Pi starvation remained fragmentary, identification of DAPs mediating its biosynthesis strongly suggests that polyamines could participate in plant adaption to P deficiency, which merits further study. 

Phosphorus deficiency also leads to the elevation of ROS in plant leaves [82]. Therefore, increased accumulations of antioxidation-related proteins were generally observed in plant leaves under P deficiency conditions, including glutathione S-transferase (GST) in maize and barley [18,23], ascorbate peroxidase (APX) in maize [18], 2-cysteine peroxiredoxin B in ramie [26], non-specific lipid-transfer protein 1, and extra-large G protein 3 in tomato [20], as well as proteins belonging to peroxidase superfamily in Arabidopsis [17]. Increased accumulations of these proteins may help plants against ROS damage under low-P stress.

### 3.2. Identification of DAPs in Plant Roots

Recently, DAPs controlling root responses to P deficiency have been identified in different plants, such as soybean [25], rape [39], and maize [19]. Functions of the DAPs have been suggested to involve a series of adaptive strategies in plant roots, including changes in root development, regulating organic acid synthesis and secretion, increasing activities of root-associated purple acid phosphatases (PAPs). Furthermore, several studies were conducted to elucidate complex responses of protein modifications to P deficiency in plant roots, especially for protein phosphorylation and succinylation.

#### 3.2.1. Pi Starvation-Responsive Proteins Participated in Root System Remodeling

**Figure 2:**
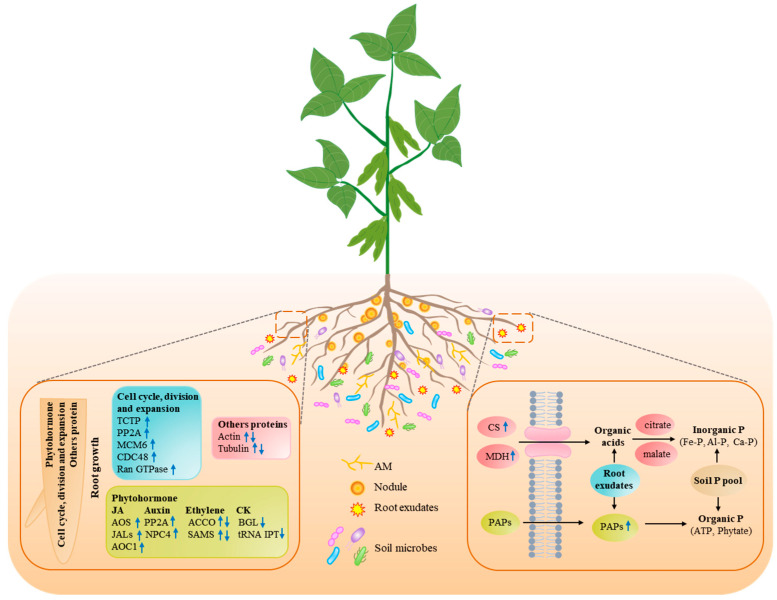
A model of integration of adaptive strategies of plant roots to Pi starvation regulated by DAPs. Up or down arrows indicate DAPs exhibiting increased or decreased accumulations; AM, arbuscular mycorrhiza; ACCO, 1-aminocyclopropane-1-carboxylate oxidase; AOC1, allene oxide cyclase 1; AOS, the allene oxide synthase; BGL, beta-glucosidase; CDC48, cell division cycle protein 48; CS, citrate synthase; JALs, jacalin-related lections; MCM6, mini-chromosome maintenance protein 6; MDH, malate dehydrogenase; PAPs, purple acid phosphatases; PP2A, phosphatase 2A; NPC4, non-phospholipase C4; Ran GTPase, GTP-binding nuclear protein RAN-B1; SAMS, S-adenosyl methionine synthase; TCTP, translationally controlled tumor protein; tRNA IPT, tRNA isopentenyl transferase.

It is well known that root architecture remodeling involves changes in hormones, such as auxin, ethylene, cytokinin (CK), or jasmonic acid (JA), which react differently but coordinately regulate root growth [83,84,85,86]. Thus, a set of DAPs was found to involve hormone synthesis, transport, and distribution in root response to P deficiency [19,31,32,33,34,35] (Figure 2). For example, in several plant species, such as rice and masson pine (*Pinus massoniana*), comparative proteomic studies were conducted to find that abundance of several ethylene-precursor synthetases, including 1-aminocyclopropane-1-carboxylate oxidase (ACCO) and SAMS, was up-regulated by P deficiency, even though ACCO and SAMS were down-regulated in maize and Arabidopsis, respectively [30,33,34,42]. The results strongly suggest that ethylene participated in regulating root growth under P-deprivation conditions, which was probably attributed to changes in its biosynthesis [87,88]. Consistently, in Arabidopsis, the number of lateral roots was significantly higher in *aco1-1* mutant compared to that in wild-type, strongly suggesting that ethylene could regulate lateral root development [89]. In addition to ethylene, auxin is considered to play a key role in mediating the development of lateral root and root hair under Pi-starvation conditions [85,90]. Consistently, a set of DAPs for the auxin signaling pathway was observed to be up-regulated in Arabidopsis and maize, including auxin-response proteins (ARFs), phosphatase 2A (PP2A), and nonspecific phospholipase C4 (NPC4) [16,19,32,41]. Furthermore, overexpression of *ZmPP2AA1*, encoding a subunit of phosphatase 2A, significantly increased the lateral root density and promoted root growth under P-deficiency conditions in maize, strongly suggesting that auxin could regulate lateral root development [91,92]. Additionally, root growth in response to P deficiency was also found to be mediated by the JA pathway, supported by the evidence that allene oxide synthase (AOS), allene oxide cyclase 1 (AOC1), and jacalin-related lections (e.g., JAL5/23/31) for controlling JA metabolism were up-regulated in Arabidopsis under low-P conditions [17,75]. Interestingly, knockout of *atwrky6-1*, a mediator in the Pi-signaling network, led to decreases in lateral root formation, but increased accumulations of AOS, strongly suggesting the JA metabolism might participate in regulating Arabidopsis root growth [17,93]. Meanwhile, decreased accumulations of tRNA isopentenyl transferase (tRNA IPT) and beta-glucosidease (BGL) involved CK synthesis were observed in long-term P-deficient maize roots, suggesting that CK could regulate plant root growth [31,32]. Moreover, several studies reported that the application of CK could disturb the expression of *PIN* genes and prevent the formation of the auxin gradient, which is required for lateral root primordia development. In contrast, the biosynthesis of CK was rapidly suppressed by auxin via the isopenteneyadenosine-50-monophosphate independent pathway [94,95,96]. Several studies also reported that ethylene could regulate root growth via stimulating auxin biosynthesis and transport in root apex, but DAPs involved in both auxin- and ethylene-signaling pathways were scarcely identified [96,97,98]. These results suggest that P deficiency-induced changes in root morphology and architecture may be modulated through sophisticated interactions among different phytohormones.

In addition to the identification of phytohormone-related DAPs, a set of DAPs involved in cell cycle, division, and expansion processes was also suggested to regulate root architecture plasticity in response to low-P stress [99,100] (Figure 2). For example, translationally controlled tumor protein (TCTP), functioning in fundamental biological processes (e.g., mitotic, cell proliferation, cytoprotective, and anti-apoptotic), was observed to be up-regulated by Pi starvation in Arabidopsis [40,101,102]. Further genetic analysis showed that reduced *AtTCTP1* expression could result in significant inhibition of lateral root formation [103]. Additionally, accumulations of cell proliferation-related proteins, including GTP-binding nuclear protein RAN-B1 (Ran GTPase), cell division cycle protein 48 (CDC48), and mini-chromosome maintenance protein 6 (MCM6), were increased by Pi starvation in maize [19,31]. Contrastingly, an increased abundance of actin and tubulin was observed in plants in response to low-P stress in plants, including soybean, maize, rice, and barley, while decreased abundance was observed in Arabidopsis [19,23,31,35,36]. Interestingly, a loss of *rmd* mutant, encoding a rice actin-binding protein, exhibited steeper growth angles for crown roots in rice, strongly suggesting that cell proliferation-related proteins could play a role in root growth [104].

#### 3.2.2. Pi Starvation-Responsive Proteins Related to Root Exudates

Plant root exudates mainly include a class of metabolites and proteins with low- or high-molecular weight, such as organic acids (OAs), sugars, flavonoids, and phosphatases [100,105]. It is well known that root exudates play crucial roles in increasing Pi uptake by desorbing immobile P in soils [100,106]. Consistently, differential proteomic analysis led to identifying a set of DAPs controlling root exudates, which contributes to deepening understandings of the molecular mechanisms underlying rhizosphere P mobilization [100,105] (Figure 2). For example, increased OA exudation was found to be associated with activities of several differential proteins related to OA synthesis, including citrate synthase (CS) in Arabidopsis, maize and soybean, IDH in maize, MDH in rape, soybean, and maize, as well as decreased abundance of aconitase in rice [21,30,32,35,41]. Consistently, overexpression of *CS* from *Daucus carota* (*DcCS*) improved Arabidopsis growth under P deficiency conditions due to enhanced citrate synthesis and exudation from the roots [107]. These results strongly indicate that increased OA synthesis and exudation play an important role for plants when scavenging Pi in soils.

In addition to OA synthesis and exudation, some secreted proteins, such as purple acid phosphatases (PAPs) and ribonucleases, are critical for plants to utilize organic-P pools in soils. Among PAPs, root-associated PAPs were widely found to be involved in the activation and utilization of extracellular organic-P sources, such as ATP, deoxy-ribonucleotide triphosphate (dNTPs), and phytate-P [56,108]. For example, several PAPs were purified and functionally characterized to mediate organic-P utilization, such as SgPAP23 in stylo (*stylosanthes guianensis*), GmPAP7a/b in soybean, OsPAP10a in rice, and LeSAP1/2 in tomato [25,47,109,110,111]. Meanwhile, a variety of low P-induced PAPs was identified in plants through proteomic analysis, such as AtPAP12/26 in Arabidopsis, GmPAP1-like, and GmPAP22-like proteins in soybean [25,44]. In Arabidopsis, *atpap12*/*atpap26* mutant exhibited impaired growth coupling the decreases in root secretory APase activity and total Pi concentration in rosettes [112,113]. Overexpressing *GmPAP1-like* enhanced Arabidopsis growth and P content when dNTP was supplied as the sole external P source [25]. In addition, some DAPs functions in cell wall carbohydrate metabolism were also identified to be up-regulated in Arabidopsis suspension cells or root cell walls under P-deficient conditions, such as polygalacturonase (PG) and xyloglucan endotransglycosylase (XET), which was suggested to release cell wall-bound Pi under low-P conditions [25,112,114]. Additionally, since changes in polysaccharides in cell wall metabolism could influence the rhizosphere size, which was suggested to be positively correlated with P accumulation in plants, DAPs related to cell wall metabolism might affect plant P efficiency partially through changing rhizosphere size [115].

#### 3.2.3. Response of Symbiotic Association to Pi Starvation in Plants

Although it is well known that the formation of a symbiotic association between plants and arbuscular mycorrhiza (AM) is a typical plant strategy in response to P deficiency, no proteomic analysis was conducted to identify DAPs in the symbiotic association at different P levels. However, several studies have highlighted that Pi acquisition efficiency in legume crops could be improved through formation of the symbiotic association between roots and rhizobia, which can directly fix atmosphere N_2_ in nodules and supply nitrogen for plant growth. For example, rhizobium inoculation led to enhancing the capability of soybean in utilization of sparingly soluble P forms (e.g., Ca-P, Al-P, Fe-P), as reflected by higher P content and plant biomass [116]. Furthermore, overexpression of two nodule preferring Pi transporters, *GmPT7* and *GmPT5* promoted soybean nodulation, P content, as well as fresh weight [117,118]. To date, only one study has elucidated differential protein profiles in soybean nodules at two P levels through two-dimensional electrophoresis combined with matrix-assisted laser desorption ionization (MALDI)-time of flight (TOF)/TOF mass spectrometry (MS) analysis. A total of 44 DAPs were identified in soybean nodules, which were mainly involved in stress response and carbon and amino acid metabolism, strongly indicating that a complex but precise module exists in soybean nodules to sustain mutualistic symbiosis to maximize Pi uptake [24]. Among them, GmMDH12 was found to be up-regulated in soybean nodules by Pi starvation, which was consistent with the increased malate concentration in nodules at low-P levels. Furthermore, overexpression of *GmMDH12* significantly increased malate concentrations and inhibited nodule size in soybean, strongly suggesting that Pi-starvation increased malate, which might have multiple functions except for the enhancement of malate exudation and the activation of sparingly soluble P in soils [119].

## 4. Identification of DAPs Exhibiting Post-Transcriptional Modifications

Post-transcriptional modifications (PTMs) play crucial roles in regulating protein activity, longevity, and localization, including phosphorylation, ubiquitination, succinylation, glycosylation, and SUMOylation modification [15,38,120]. Recently, several studies were conducted to investigate changes in protein profiles with PTMs in plant roots at different P levels, such as phosphoproteome in soybean, maize, and rice [15,22,38]. In soybean roots, a total of 427 phosphoproteins were found to be regulated by Pi starvation, including 213 up-regulated and 214 down-regulated [15]. For example, auxin efflux transporter GmPIN2 and ethylene-insensitive protein GmEIN2 were identified to be up-regulated by P deprivation, strongly suggesting participation of auxin and ethylene in modulating root architecture in response to P deficiency [15]. Meanwhile, in rice roots, a total of 554 phosphoproteins were found to exhibit differential accumulations, with 546 down-regulated and 8 up-regulated proteins. Several proteins, including four mitogen-activated protein kinases (MAPKs), five calcium-dependent protein kinases (CDPKs), and OsCK2β3, were observed to be decreased in response to Pi starvation, especially for OsCK2, which was suggested to regulate phosphorylation of OsPT2 and OsPT8 and thus influence their subcellular localization [22,121]. Meanwhile, in maize roots, about 51 phosphoprotein spots were observed to exhibit differential accumulations by low-P treatment; these were involved in a group of cellular and metabolic pathways, such as signal transduction and carbon metabolism [32]. Among them, one auxin receptor, ABP1, was found to be up-regulated in maize roots under low-P conditions [32]. Recently, overexpression of *AtABP1* has been found to influence primary root growth, root bending, and lateral root development [122]. 

In addition to phosphorylation analysis, succinylated-proteomic analysis was also conducted to identify differential proteins with succinylation in barley roots under P-deficiency conditions [38]. A total of 120 succinylation sites were identified across 83 proteins, including 79 increased and 4 decreased succinylated proteins at 48 h of Pi-starvation treatment, respectively [38]. Moreover, these differentially succinylated proteins were enriched in ribosome pathways, glycolysis, and RNA degradation pathways. For example, 60S/50/40S/30 ribosomal protein was suggested to involve ribosome pathways, while TPI, GAPDH, and FBP aldolase were predicted to participate in glycolysis pathways [38]. However, functions for most of the identified proteins with succinylation remain unknown, which merits further study.

## 5. Perspectives

In recent decades, with the aid of proteomic analysis, identification and functional characterization of DAPs have opened the way to elucidate molecular mechanisms underlying plant adaptation to Pi starvation. For example, the increased abundances of SPX1, PHT1;4, and PHF1 proteins were observed in Arabidopsis roots, and other proteins, such as TaPHT1;9-4B, TaPHT1;3-5B, and TaPHT1;6-5B, were also observed in wheat roots, acting as the main proteins mediating Pi homeostasis [16,37]. However, the functions of most of the DAPs remain largely unknown and require furthered investigation via reverse and forward genetic analysis. For example, a histone chaperone (*nap1;2*) was found to be up-regulated under Pi starvation via proteomic analysis in Arabidopsis, and its functions in maintaining Pi homeostasis were clarified via analysis of triple *nap1;1 nap1;2 nap1;3* mutant lines [13]. Meanwhile, although the method of differential proteomics based on mass spectrometry was established and successfully used, there is still a lack of a more effective method for protein separation and identification in order to qualitatively analyze plant proteins. In order to solve these problems, multiple technologies should be combined to develop a more effective method for protein extraction and separation. Meanwhile, in addition to commonly used quantitative techniques, such as label-free quantitation [123], isobaric tags for relative and absolute quantitation (iTRAQ) [124], tandem mass tags (TMT) [125], and stable isotope labeling by amino acids in cell culture (SILAC) [126], application of a relatively new technique, termed sequential window acquisition of all theoretical mass spectra (SWATH) [127], together with data-independent acquisition (DIA) [128], might be very helpful to identify more DAPs. Furthermore, the vigorous development and wide application of new technologies (e.g., microfluidic chip, reverse micelles, magnetic nanoparticles) will break the bottleneck of protein separation, enrichment, and detection in single cells and different organelles [129].

Although many questions remain to be solved, we believe that the application of proteomics integrating multiple omics, as well as genetic analysis, will help us to elucidate molecular mechanisms underlying plant adaptation to P deficiency and develop cultivars with high P efficiency in the future.

## Figures and Tables

**Table 1:** A list of proteomic analyses of plant responses to phosphorus deficiency.

Plant Species	Organ/Tissues	Culture Time before Treatment (d)	Treatment Time (d)	Protein Separation Method	Total Protein Number (#)	Number of DAPs (#)	Protein Number Identified by MS Analysis		References
							Up-Regulated	Down-Regulated	
*Arabidopsis thaliana*	Leaves	10	7	SCX iTRAQ LC-MS/MS	5106	156	106	50	[17]
*Zea mays*	Leaves	4	25	2-DE MALDI TOF MS/TOF	1342	200	nd	nd	[26]
*Zea mays*	Leaves ^a^	18	25	2-DE MALDI-TOF/MALDI-TOF-TOF MS	680/592	29/71	9/20	55/16	[27]
*Glycine max*	Leaves	5	14	2D-IEF/SDS-PAGE MALDI-TOF MS	55	17	7	10	[28]
*Glycine max*	Leaves	3	14	SDS-PAGE Gel Digestion LC-MS/MS	4219	707	267	440	[29]
*Solanum lycopersicum*	Leaves	nd	10	2-DE MALDI-TOF MS/MS/MS	600	46	31	15	[20]
*Arabidopsis thaliana*	Roots	10	3	2-DE MALDI TOF MS	456	30	nd	nd	[30]
*Arabidopsis thaliana*	Roots	10	3	2-DE iTARQ LC-MS	13,298	356	199	157	[16]
*Arabidopsis thaliana*	Roots	nd	14	2-DIGE MALDI-TOF/TOF	1420	30	14	16	[13]
*Zea mays*	Roots	24	17	2-DE MALDI TOF MS	1300	254	76	30	[31]
*Zea mays*	Roots	10	1/3/7/11	2-DE MALDI-TOF-MS	850	91	nd	nd	[32]
*Zea mays*	Roots ^a^	24	17	2-DE MALDI TOF MS	2822	73/95	25/24	12/6	[19]
*Zea mays*	Roots ^a^	nd	10	2-DE MALDI-TOF	nd	83/325	30/246	53/79	[33]
*Oryza sativa*	Roots	3	80	2-DE MALDI TOF MS	669	34	nd	nd	[21]
*Oryza sativa*	Roots	7	21	2-DE MALDI-TOF MS	140	10	2	8	[34]
*Glycine max*	Roots	nd	20	2-DIGE	325	105	61	44	[35]
*Glycine max*	Roots ^a^	3	9	SDS-PAGE TMT	4216	660/133	656/127	4/6	[36]
*Glycine max*	Roots	nd	10	iTRAQ LC-MS/MS	nd	71	30	41	[25]
*Glycine max*	Roots	5	14	iTRAQ	nd	427	213	214	[15]
*Triticum aestivum*	Roots	14	8	iTRAQ	6842	323	nd	nd	[37]
*Hordeum vulgare*	Roots	10	0.25/2	SDS PAGE LC-MS/MS	nd	697	nd	nd	[38]
*Brassica napus*	Roots ^a^	20	3	2-phase LC/MS-MS	828	31/40	8/28	23/12	[39]
*Arabidopsis thaliana*	Leaves/roots	7	3	2-DIGE MALDI TOF/TOF MS	88	nd	nd	nd	[40]
*Hordeum vulgare*	Leaves/roots	17	21	2-DE MALDI-TOF/TOF-MS	nd	31	nd	nd	[23]
*Brassica napus*	Leaves/roots	20	26	2-DE MALDI TOF MS	1000	32	4/12	13/3	[41]
*Pinus massoniana*	Seedlings	10	58	2-DE MALDI-TOF/TOF MS	nd	98	44	54	[42]
*Arabidopsis thaliana*	Suspension cells	7	7	2-DE MALDI TOF MS	110	46	26	6	[43]
*Arabidopsis thaliana*	Suspension cells	9	2	SDS-PAGE LFQ	5013/1881	1169/994	nd	nd	[44]
*Glycine max*	Nodules	5	25	2-DE MALDI TOF MS	nd	44	17	27	[24]

^a^ Two genotypes were used in the studies. nd, not described.
